# Research Status and Latest Progress in the Regulatory Mechanisms of ABCA1

**DOI:** 10.3390/ijms262210855

**Published:** 2025-11-08

**Authors:** Xingtong Chen, Yunyue Zhou, Jinbiao Yang, Shuang Xue, Qiao Wang, Xuan Guo, Yukun Zhang, Wenying Niu

**Affiliations:** School of Basic Medical Sciences, Heilongjiang University of Chinese Medicine, Harbin 150040, China; chenxingtong@hljucm.edu.cn (X.C.);

**Keywords:** ABCA1, cholesterol efflux, advances in research

## Abstract

Cholesterol is an essential lipid in the human body, involved in critical physiological processes such as cell membrane composition and hormone synthesis. The homeostasis of cholesterol is vital for the normal functioning of the organism. Reverse Cholesterol Transport (RCT) is a core mechanism maintaining this balance, and ABCA1, as a key membrane transporter, plays a decisive role in RCT by mediating cholesterol efflux to HDL precursors, thereby promoting the initial formation of HDL. The regulatory mechanism of ABCA1 is extremely complex, with its regulation mainly occurring through two dimensions: transcriptional expression and post-translational modification. Currently, clinical drugs for regulating cholesterol are dominated by statins, supplemented by ezetimibe, PCSK9 inhibitors, and others. However, these drugs have certain limitations, and research on ABCA1-targeted drugs is relatively scarce. Therefore, summarizing the research progress on the regulatory mechanism of ABCA1 is expected to provide important insights for the development of new therapies to maintain cholesterol homeostasis.

## 1. Introduction

**Figure 1:**
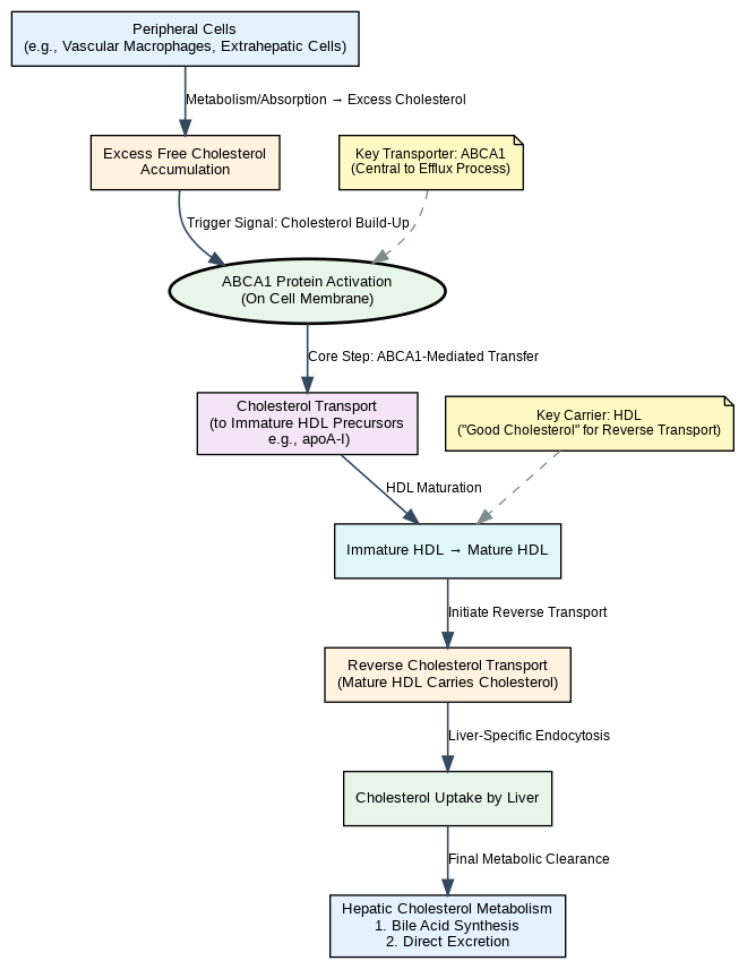
Involvement of ABCA1 in the RCT Process.

Cholesterol, a member of the steroid family, is one of the important lipid substances in the human body. It serves as a crucial component of eukaryotic cell membranes and acts as a precursor for bile acids and steroid hormones. It is essential for embryonic development and cell proliferation, and can also be covalently modified onto certain proteins to participate in specific signal transduction processes. The cholesterol level in the human body is influenced by multiple factors, including intracellular cholesterol content, energy supply (ATP availability), and hormone levels. “Reverse Cholesterol Transport (RCT)” is one of the core mechanisms maintaining the balance of cholesterol metabolism in the human body. It mainly refers to the process by which peripheral tissues (such as macrophages in the vascular wall and cells of other organs outside the liver) transport excess cholesterol to high-density lipoprotein (HDL), which then carries it back to the liver for metabolic excretion. As shown in Figure 1. ABCA1 (ATP-Binding Cassette Subfamily A Member 1) is a key protein in the cholesterol efflux process. Belonging to the ATP-binding cassette (ABC) transporter superfamily, ABCA1 is localized on cell membranes (e.g., those of macrophages, hepatocytes, and intestinal epithelial cells). Its primary function is to transport “free cholesterol” on the cell membrane to immature HDL precursors, thereby promoting the initial formation of HDL [1,2]. Abnormal expression of ABCA1 is closely associated with the development and progression of various diseases. Reduced ABCA1 expression in macrophages and vascular endothelial cells exacerbates cholesterol deposition in the arterial wall, promoting the formation and progression of atherosclerotic plaques [3]. The deficiency of ABCA1 decreases cholesterol secretion to exogenous ApoE, leading to cholesterol accumulation in astrocytes and contributing to the occurrence of Alzheimer’s disease [4]. A large number of studies have reported that ABCA1 is related to the mechanism, metastatic ability, and prognosis of various cancers [5]. It is evident that dysregulated cholesterol metabolism caused by abnormal ABCA1 expression is one of the common pathological bases for multiple diseases. Therefore, how to achieve cholesterol homeostasis in the body has become a current research focus. Currently, there are no clinical drugs targeting ABCA1 to improve cholesterol efflux. The commonly used cholesterol-regulating drugs are still mainly statins (which inhibit cholesterol synthesis). In addition to statins, there are also drugs such as ezetimibe (a cholesterol absorption inhibitor), PCSK9 inhibitors, and cholestyramine (a bile acid sequestrant) that can regulate cholesterol through different pathways. Specifically, ezetimibe affects cholesterol absorption, PCSK9 inhibitors promote the liver’s clearance of low-density lipoprotein cholesterol (LDL-C) from the blood, and cholestyramine facilitates the conversion of cholesterol into bile acids while preventing the reabsorption of bile acids. However, these drugs are less commonly used in clinical practice compared to statins, and in many cases, they are used in combination with statins. Nevertheless, an increasing number of reports indicate that these drugs exhibit side effects of varying degrees, and at the same time, there are relatively few studies on drugs targeting ABCA1-related targets [6]. Thus, research on the regulatory mechanisms related to ABCA1 may provide new directions and ideas for the development of drugs targeting cholesterol homeostasis.

## 2. ABCA1 Structure and Intracellular Trafficking

The human *ABCA1* gene is localized at chromosome 9q31, with a total length of 149 kb, encompassing 50 exons and 49 introns. The ABCA1 protein is an integral membrane protein consisting of 2261 amino acids, composed of two symmetric transmembrane domains (TMDs). Each TMD contains a tandem repeat sequence of 6 transmembrane segments (TMSs) and 1 nucleotide-binding domain (NBD). The NBD, serving as the ATP-binding site, is composed of two peptide sequences designated as Walker A and Walker B, which provide the energy required for substance transmembrane transport. The membrane topology of ABCA1 reveals that it includes a cytoplasm-facing N-terminus and two extracellular loops. These extracellular loops are highly glycosylated and linked by one or more cysteine residues, a feature associated with the specific binding to apolipoprotein A I (apoA I) [7,8].

The intracellular trafficking mechanism of human ABCA1 exhibits a high degree of complexity. ABCA1 initiates its synthesis within the endoplasmic reticulum (ER). During and after synthesis, it undergoes a series of post-translational modifications and structural maturation processes: this includes N-glycosylation (where oligosaccharide residues are attached), the formation of dimers, the establishment of disulfide bonds between its two extracellular domains (ECDs), and the achievement of correct protein folding. Following the completion of these maturation steps, ABCA1 is transported to the Golgi apparatus by means of vesicular carriers. From the Golgi apparatus, it is then further routed to the plasma membrane to fulfill its biological functions [9]. The transport process of ABCA1 from the ER to the Golgi apparatus involves vesicles coated with coat protein II (COPII) [10]. Studies have shown that BIG1 (brefeldin A-inhibited guanine nucleotide-exchange protein 1) and Rab8 have been identified to function in regulating the trafficking of ABCA1 to the cell membrane. However, current research on the mechanisms controlling ABCA1 trafficking remains limited and unclear [11,12,13].

## 3. Factors Related to Affecting the Transcriptional Expression of ABCA1

### 3.1. LXRα/ABCA1 Pathway

As a key molecule for maintaining cellular lipid homeostasis and RCT, the transcriptional expression of ABCA1 is precisely controlled by multi-dimensional regulatory mechanisms. Among these mechanisms, the LXRα/ABCA1 pathway is one of the most in-depth studied pathways and plays a core regulatory role in ABCA1 transcription. Liver X Receptors (LXRs), including the LXRα and LXRβ subtypes, are essential oxysterol-activated transcription factors involved in lipid metabolism and immune responses. LXRα is highly expressed in the liver, adipose tissue, and macrophages, while LXRβ is ubiquitously expressed. In macrophages, LXRα directly promotes RCT by upregulating the transcriptional expression of ABCA1; therefore, LXRα deficiency impairs cholesterol efflux [14]. When the intracellular concentration of free cholesterol increases (e.g., when macrophages phagocytose lipids to form foam cells, or hepatocytes take up excessive cholesterol), cytochrome P450 enzymes (such as CYP46A1 and CYP27A1) catalyze the conversion of cholesterol into “endogenous activating ligands” of LXRs, including oxysterols (e.g., 24(S)-OHC and 27-OHC). These oxysterols enter the nucleus through diffusion and specifically bind to the ligand-binding domain of LXRs. After ligand binding, LXRs undergo a critical conformational change, switching from an “inactive state” to an “active state”. However, LXRs cannot bind to DNA or initiate transcription independently; they must form LXR-RXR heterodimers with another nuclear receptor, retinoic acid X receptor (RXR), which is a prerequisite for their exertion of transcriptional regulatory function. Subsequently, the DNA-binding domain (DBD) of the LXR-RXR heterodimer undergoes “sequence-specific binding” to the LXR response element (LXRE) sequence of ABCA1, thereby initiating subsequent transcriptional activation [15]. Peroxisome proliferator-activated receptor γ (PPARγ) is a key receptor for adipocyte differentiation and lipid metabolism. It can either directly bind to the “PPAR response element (PPRE)” in the ABCA1 promoter or upregulate the expression of LXRs to enhance the binding ability of LXR-RXR to LXREs, thereby further upregulating ABCA1 expression [16]. In addition, studies have reported that the absence of SR-B1 (scavenger receptor class B type 1)-mediated cholesterol movement in the liver does not affect the expression of major genes involved in hepatic cholesterol flux. However, FXR activation stimulates intestinal cholesterol excretion, upregulates the expression of ABCA1, ABCG5, and ABCG8 in the ileum to varying degrees, and reduces diet-induced hyperlipidemia by increasing the expression of ileal cholesterol transporters. Nevertheless, the specific mechanism by which FXR upregulates ABCA1 has not been clarified [17].

Currently, research on the regulation of ABCA1 transcriptional expression mainly focuses on the use of “LXRα activators” to enhance ABCA1 transcriptional expression. For example, T0901317 is a highly potent LXR agonist that can cross the blood–brain barrier and effectively activate LXRs. However, studies have shown that it lacks specificity and cannot specifically activate LXRα, thus its application is subject to certain limitations [18]. In addition, targeting DHCR24 (24-dehydrocholesterol reductase) to increase endogenous desmosterol levels has been recognized as a promising strategy to activate the LXR transcriptional program, enhance ABCA1-mediated cholesterol efflux, and counteract atherosclerotic cardiovascular diseases [19]. Exogenous Toll-like receptor 2 (TLR2) can reduce cholesterol efflux by decreasing the expression of ABCA1, ABCG1, and SR-B1 in a dose-dependent manner. The specific mechanism may be that blocking TLR signaling downregulates p65 phosphorylation, thereby activating the PPARγ-LXRα-ABCA1/ABCG1/SR-B1 pathway [20]. It was previously reported that SREBP-2 (sterol regulatory element-binding protein 2) can bind to the E-box element in the proximal promoter of ABCA1, leading to the downregulation of ABCA1 expression. However, Wong et al. verified through experiments that this is not the case; the results showed that SREBP-2 plays a key role as a positive regulator of ABCA1 gene expression by generating oxysterol ligands for LXRs [21]. Besides the aforementioned factors, a large number of other factors such as Platycodin D, lycopene, Allyl isothiocyanate, CTRP9 (C1q/tumor necrosis factor-related protein 9), and immunoglobulin-like type 2 receptor β can also regulate the LXRα/ABCA1 signaling pathway and modulate ABCA1 expression [22,23,24,25,26].

The expression of ABCA1 is not only regulated by the LXRα/ABCA1 pathway but also subject to transcriptional regulation through multiple processes, such as: JAK2/STAT3 Pathway: STAT3 possesses the ability to bind to the specific promoter region of the ABCA1 gene. Through this targeted binding interaction, STAT3 exerts a regulatory effect that ultimately enhances the expression level of the ABCA1 protein. The JAK family is the most important factor for activating the phosphorylation of STAT3. When JAK2 activates STAT3, the expression of ABCA1 in macrophages is induced and enhanced [27,28]. NF-κB Signaling Pathway: Studies have shown that NF-κB can downregulate ABCA1/G1 (ABCA1 and ABCG1) expression. NF-κB mediates the activation of SREBP-2 and miR-33a expression. However, the specific crosstalk mechanism between chronic inflammation and NF-κB-mediated changes in SREBP-2, miR-33a, and ABCA1/G1 remains unclear [29]. PI3K/AKT Pathway: Research reports indicate that activation of the PI3K/AKT (phosphatidylinositol 3-kinase/protein kinase B) pathway can promote the expression of FoxO1 (forkhead box O1). FoxO1 inhibits the activity of the ABCA1 promoter, thereby reducing ABCA1 expression. Meanwhile, there are also reports stating that activation of PI3K/AKT can upregulate ABCA1 expression, but the specific mechanism has not been clarified [30,31]. In addition, some common signaling pathways such as the PKC (protein kinase C) pathway and RAR-RXR pathway have all been mentioned in reports to be capable of upregulating ABCA1 expression [27,32].

### 3.2. MicroRNA (miR)

MicroRNAs (miRNAs) represent a category of evolutionarily conserved, endogenous small non-coding RNAs with important functional roles. These molecules are transcribed from distinct genomic regions, including intergenic segments, introns, or exons of protein-coding genes, and undergo stepwise processing via either canonical pathways [33] or non-canonical pathways [34]. Mature miRNAs, which range in length from 17 to 25 nucleotides, typically interact with the 3′ untranslated region (3′ UTR) of their target messenger RNAs (mRNAs). This interaction is characterized by partial or complete complementarity between the miRNA’s seed region—the 2-8 nucleotide sequence located at the 5′ end of the miRNA—and the target mRNA sequence. When associated with the RNA-induced silencing complex (RISC), mature miRNAs trigger either degradation of the target mRNA or repression of its translation; both outcomes ultimately lead to downregulated expression of the corresponding target gene [35]. In a specific study, Chowdhari et al. identified that the miRNA encoded by hepatitis B virus (HBV), known as HBV-miR-3, can directly target the ABCA1 gene. Experimental data from their work demonstrated that HBV-miR-3 inhibits ABCA1 expression, promotes the intracellular accumulation of cholesterol, and disrupts the normal lipid metabolism of hepatocytes. These findings thus highlight the potential role of the HBV-miR-3-ABCA1 regulatory axis in the development of hepatocellular carcinoma [36]. Additionally, previous reports have indicated that inhibition of the Notch signaling pathway can result in suppressed ABCA1 expression. Notably, miR-200b-3p and miR-424-5p have been suggested to downregulate the expression of Notch1 and Notch2—key components of the Notch signaling pathway. By modulating these Notch proteins, these miRNAs help regulate lipid homeostasis and inhibit the progression of atherosclerosis. Furthermore, atmospheric particulate matter (PM2.5) has been shown to reduce the expression levels of miR-200b-3p and miR-424-5p, which in turn facilitates the development of atherosclerosis [37].

miR-33a, an intronic microRNA encoded within SREBP-2 (sterol regulatory element-binding protein 2), is widely recognized as a key regulator of cholesterol metabolism and homeostasis [38]. Similar to other microRNAs, miR-33a is processed into two mature strands, namely miR-33a-5p and miR-33a-3p. A large number of studies have shown that miR-33a-5p effectively silences ABCA1 expression, and the expression of miR-33a-5p plays a certain role in atherosclerotic diseases. In contrast, research on miR-33a-3p is relatively limited; although some studies have reported that it can inhibit ABCA1 expression, no detailed investigation has been conducted [39]. For example, researchers found that targeted suppression of miR-33a-3p on its own does not increase cholesterol efflux dependent on ABCA1 in proinflammatory endothelial cells [40]. This indicates that additional anti-atherosclerotic strategies targeting miR-33a-3p are required, and whether it can regulate ABCA1 expression awaits systematic and comprehensive exploration. Ou et al. demonstrated through studies that miR-223 positively regulates ABCA1 expression, and it is speculated that miR-223 may promote ABCA1 expression through multiple indirect mechanisms. Among these, the most extensively studied mechanism is that miR-223 inhibits its direct target gene Sp3, thereby indirectly promoting ABCA1 expression [41]. In addition to the aforementioned microRNAs, numerous current studies have confirmed that there are other microRNA molecules capable of regulating ABCA1 expression. Among them, represented by miR-144-3p, miR-26a-5p, miR-128, miR-148a-3p, and miR-96-5p, relevant studies have clearly defined their regulatory effects on ABCA1 expression levels. This provides important molecular target references for further exploring the physiological and pathological processes mediated by ABCA1 [42,43,44,45,46].

### 3.3. Non-Coding RNA (ncRNA)

Non-coding RNAs (ncRNAs) constitute a broad category of RNA molecules that lack the ability to encode proteins. These molecules exert vital functions in nearly all biological processes, encompassing the regulation of gene expression, cellular metabolic activities, and developmental differentiation events. A distinct subset within the ncRNA family is referred to as circular RNAs (circRNAs). CircRNAs are defined by their unique covalently closed loop structure, which is formed through a specialized back-splicing mechanism [47]. Functionally, circRNAs can modulate gene expression in two main ways: either by acting as competing “sponges” for microRNAs (miRNAs) or by interacting with RNA-binding proteins. In one research study, Wu and his team identified that miR-23a-5p is capable of binding to the 3′ untranslated region (3′ UTR) of circ8411. This binding interaction inhibits the functional activity of circ8411, which in turn regulates the expression level of the ABCA1 gene [47]. In a separate investigation, Xu et al. explored the role of circRNAs in atherosclerosis and uncovered that circRNA circDENND1B serves as a promising novel mediator of atherosclerosis in mouse models. The expression level of circDENND1B shows a negative correlation with both the progression of atherosclerosis and the formation of foam cells. Notably, upregulating circDENND1B expression significantly reduces ox-LDL-induced foam cell formation by enhancing the process of cholesterol efflux. The specific underlying mechanism involves circDENND1B acting as a sponge for mmu-miR-17-5p. By sequestering this miRNA, circDENND1B promotes the increased expression of Abca1 in cells that have been treated with IL-1β monoclonal antibody (IL-1β mAb) [48].

Long non-coding RNAs (lncRNAs) serve as crucial modulators in the process of foam cell generation and the progression of atherosclerosis (AS). Specifically, lncRNA MeXis is capable of enhancing the transcription of the ABCA1 gene. This function is achieved through its interaction with DDX17, which in turn leads to the activation of LXRα. In the context of THP-1 macrophages, when lncRNA MeXis is overexpressed, it results in elevated ABCA1 expression and further facilitates the efflux of cholesterol from macrophages [49]. Another lncRNA, DAPK-IT1, exerts an opposing effect on ABCA1. It downregulates the levels of ABCA1 by acting as a molecular sponge for miR-590-3p. This downregulation subsequently inhibits the cholesterol efflux mediated by ABCA1, ultimately contributing to a reduction in the formation of foam cells derived from THP-1 macrophages [50]. Previous studies have suggested that lncRNA DANCR possesses multiple biological functions, including the regulation of cholesterol efflux by suppressing miR-33a. However, recent research findings have revealed some unexpected results regarding the relationship between DANCR and miR-33a in THP-1 macrophages. Neither the overexpression nor the inhibition of DANCR was found to cause changes in the levels of miR-33a. Moreover, the transfection of miR-33a mimics had no impact on the influence of LV-DANCR on the expression of ABCA1/G1, SR-A, and SR-BI. Additionally, treatment with miR-33a inhibitors did not reverse the negative effect of si-DANCR on the levels of SR-A and CD36. These experimental observations collectively indicate that DANCR regulates the expression of membrane-bound cholesterol transporters through a mechanism that is independent of miR-33a. Furthermore, it was confirmed that lncRNA DANCR can effectively diminish the cholesterol efflux mediated by ABCA1/G1 and SR-BI [51]. LncRNA MALAT1 also holds significant importance in the regulation of lipid metabolism, with a particular focus on cholesterol metabolism. There exists a close regulatory association between lncRNA MALAT1 and ABCA1. Fu’s experimental results demonstrated that in chondrocytes induced by triglyceride (TG), the knockdown of lncRNA MALAT1 led to a substantial increase in the protein expression level of ABCA1. Additionally, these results provided evidence that Tougu Xiaotong Capsules can modulate the cholesterol metabolism of chondrocytes by targeting lncRNA MALAT1, thereby delaying the degenerative process of osteoarthritis [52]. AI662270, a specific lncRNA, shows a distinct distribution pattern in mouse atherosclerotic lesions. It is predominantly enriched in macrophages, while it is not detected in endothelial cells, smooth muscle cells, or fibroblasts within these lesions. Furthermore, AI662270 is upregulated in the presence of ox-LDL. In in vitro experiments, it was found to bind to ABCA1 in macrophages, and this binding is responsible for the regulation of cholesterol efflux in macrophages [53].

### 3.4. Methylation

Methylation is a key chemical modification process in organisms. Its core is the addition of a “methyl group” (composed of one carbon atom and three hydrogen atoms) to specific sites of certain molecules (such as DNA, RNA, and proteins). Without altering the basic sequence of the molecule (e.g., the base sequence of DNA), it precisely regulates the functional activity of the molecule. In 1974, scientists first discovered N6-methyladenosine (m6A) in mRNA. As a well-characterized RNA epigenetic modification, N6-methyladenosine (m6A) is widely recognized as one of the most abundant modifications in eukaryotic messenger RNAs (mRNAs). It exerts a vital regulatory function in the post-transcriptional control of gene expression [54,55,56,57]. The multi-step dynamic regulatory process of m6A modification is primarily mediated by three distinct classes of functional molecules: first, methyltransferases, which are responsible for adding methyl groups to target RNAs; second, demethylases, which carry out the demethylation of m6A-modified sites; and third, RNA-binding proteins, which specifically recognize and bind to m6A-modified regions. These regulatory factors work in a coordinated manner to participate in various mRNA-related processes, including mRNA splicing and processing, nucleocytoplasmic transport, translational regulation, and degradation metabolism [58]. The insulin-like growth factor 2 mRNA-binding protein (IGF2BP) family—comprising IGF2BP1, IGF2BP2, and IGF2BP3—has garnered extensive attention across diverse disease research fields, owing to its role as a key regulatory factor in the m6A modification process. In a specific study, Xu and his research team investigated the mechanism through which the interaction between IGF2BP1 and ABCA1 contributes to the progression of lung adenocarcinoma. To address this, they employed a range of experimental techniques, including RNA immunoprecipitation quantitative polymerase chain reaction (RIP-qPCR), Western blotting (WB) analysis, and fluorescent probe-based assays. Their findings ultimately revealed that IGF2BP1 can inhibit the expression of ABCA1 by mediating the m6A methylation modification of the ABCA1 mRNA. This regulatory effect on ABCA1 expression further impacts the molecular mechanisms governing cholesterol metabolism and, in turn, promotes the malignant progression of lung adenocarcinoma [59]. AlkB homolog 5 (ALKBH5) exerts functional effects in regulating m6A methylation modification and the development of cutaneous melanoma. ALKBH5 is an upstream target mediating the epigenetic regulation of ABCA1; it can recognize the m6A motif on the 3′ UTR of ABCA1, reduce the stability of its mRNA and the expression of its protein, thereby promoting tumor progression [60].

In addition, studies have found that the methylation of ABCA1 is also indirectly affected by several factors. For example, valeric acid, a metabolite of intestinal flora, upregulates the expression of mucin 2 (MUC2), which in turn downregulates the expression of ALKBH5 and subsequently upregulates ABCA1 expression. This process reduces intracellular cholesterol accumulation and exerts an anti-atherosclerotic effect [61]. Arsenic can promote the assembly of NADPH oxidase in THP-1 macrophages, enhance the production of ROS, and induce oxidative stress in macrophages. This thereby increases the degree of DNA methylation in the ABCA1 gene promoter region and inhibits the expression of the ABCA1 gene and protein in macrophages [62]. Wang et al. found that under the action of homocysteine (Hcy) at different concentrations, the methylation level of the ABCA1 promoter in human aortic smooth muscle cells was significantly increased, while the expression of its mRNA and protein was decreased. It is speculated that the increase in Hcy accelerates the methionine cycle, leading to an increase in DNA methyltransferases (DNMTs). The elevated DNMTs can cause hypermethylation in the promoter region of certain genes, thereby resulting in gene silencing and decreased expression [63]. EZH2 (enhancer of zeste homolog 2) can catalyze the methylation of the ABCA1 promoter region DNA and the trimethylation of histone H3K27 (lysine 27 of histone H3). This further silences the transcription and expression of ABCA1, reduces intracellular cholesterol efflux, and promotes intracellular lipid accumulation and the progression of atherosclerosis [64].

### 3.5. Acetylation

Histone deacetylases (HDACs) represent a category of protein enzymes that suppress gene transcription. Their inhibitory effect is achieved by preventing the separation of DNA from histone octamers and inducing the compaction of the nucleosome structure. Among HDACs, HDAC9 stands out as a key deacetylase, which exerts its gene transcription-inhibiting function through the process of histone deacetylation. Notably, elevated expression of HDAC9 has been detected in human carotid artery plaques, aortic plaques, and femoral plaques. In contrast, the absence of HDAC9 (HDAC9 deficiency) leads to enhanced expression of ABCA1 and ABCG1. This enhancement is driven by the promotion of acetylation at the promoters of H3, H4, and H3K9. Indole-3-carboxaldehyde (ICA), a tryptophan metabolite produced by the microbiota, has been proven to exhibit a favorable therapeutic effect on atherosclerosis. The underlying mechanism involves miR-1271-5p: this microRNA activates the miR-1271-5p/HDAC9 signaling cascade, and this activation ultimately results in increased ABCA1 expression [65]. Another compound, berberine, is capable of promoting ABCA1 expression in foam cells derived from THP-1 macrophages. Additionally, it facilitates the efflux of intracellular cholesterol that is mediated by ABCA1. Importantly, this regulatory effect of berberine is associated with its ability to inhibit the acetylation of LXR-α [66].

### 3.6. Other Factors

The transcriptional expression of ABCA1 is also affected by other factors. For example, studies have reported that related proteins that can bind to its promoter, cytokines, hormones, and other substances all play a role in regulating the transcriptional expression of ABCA1. The transcriptional expression of ABCA1 is mainly mediated through the conserved consensus cis-acting element DR4, which is the binding site of the nuclear receptor LXRα in the proximal promoter region (TGACCGatagTAACCT) of the ABCA1 gene. After activation, LXRα forms a heterodimer with its partner protein RXR and binds to the DR4 site, thereby increasing the transcription of the ABCA1 gene. Currently, many substances have been reported to be able to bind to DR4 and regulate ABCA1. For instance, niacin can enhance ABCA1 transcription [67], while oxysterol-binding protein-related protein 8 (ORP8) can inhibit ABCA1 expression and cholesterol efflux in macrophages [68].

Zinc finger protein 202 (ZNF202) functions as a key transcriptional repressor, with the ability to bind to specific promoter elements—elements that are predominantly found in genes involved in lipid metabolism pathways. Langmann et al. carried out an in-depth analysis of the transcriptional regulation mechanisms of the ZNF202 gene. Their research ultimately demonstrated that the ZNF202/SRE-like binding motif, located within the ABCA1 promoter region, controls the tissue-specific expression of ABCA1. This control is achieved through the motif’s site-specific binding to ZNF202. Additionally, their findings revealed a mutually dependent negative correlation between the expression levels of ZNF202 and ABCA1 [69]. Separate studies have identified that the further enhancement of ABCA1 transcriptional activity is mediated by the -175 bp segment of its promoter region. In vitro experiments showed that the transcription factor Sp1 binds to the -91 GnC motif within this promoter, while both Sp1 and Sp3 can bind to the -157 GnC promoter region. Functional assays indicated that overexpression of Sp1 leads to increased ABCA1 mRNA expression in HeLa cells; in RAW246.7 macrophages, this overexpression also enhances the efflux of cellular cholesterol and phospholipids. In contrast, Sp3 exerts an inhibitory effect on ABCA1 transcription by competing with Sp1 for binding to the GnC motifs in the ABCA1 promoter [70]. Based on current research reports, the coactivators known to bind to the ABCA1 promoter include SRC1, SRC2, p300, and cAMP response element-binding protein (CREB)-binding protein (CBP) [71]. On the other hand, the corepressors involved in the transcriptional silencing of ABCA1 encompass nuclear receptor corepressor (NCoR), silencing mediator for retinoic acid and thyroid hormone receptors (SMRT), receptor-interacting protein 140 (RIP140), and small heterodimer partner (SHP) [72]. Furthermore, certain cytokines—such as interleukin-1β (IL-1β), interleukin-12 (IL-12), and interleukin-18 (IL-18)—exhibit a negative correlation with ABCA1 expression. These cytokines indirectly regulate the transcriptional expression of ABCA1, either by modulating the activity or expression of other proteins or by acting on microRNAs [73,74].

Some studies have shown that hormones are also involved in the transcription of the ABCA1 gene. Estradiol can significantly induce the level of ABCA1 mRNA in macrophages within a short period, suggesting that the ABCA1 promoter can be directly activated through estrogen receptor β (ERβ). However, whether estrogen receptors can directly activate the ABCA1 promoter has not been reported in studies [75]. The peptide hormone angiotensin II (Ang II) eliminates the anti-atherosclerotic properties of macrophages caused by LXRβ activation through the deficiency of Ang II type 2 receptor (AT2) [76]. Yang et al. found through research that Ang II increases the cholesterol content in podocytes (a component of the glomerular filtration barrier), and this change is accompanied by a decrease in the expression of ATP-binding cassette transporter 1 (ABCA1)—a molecule related to cholesterol efflux—as well as an increase in the expression of low-density lipoprotein receptor (LDLR, a molecule related to cholesterol uptake) and sterol regulatory element-binding proteins (SREBP1 and SREBP2, molecules related to cholesterol synthesis) and HMGCR. Nevertheless, methyl-β-cyclodextrin can counteract the podocyte cholesterol accumulation caused by Ang II-mediated downregulation of ABCA1 [77]. Thyroid hormone receptors (TRs) are key nuclear receptors that mediate the physiological effects of thyroid hormones (THs, mainly including T3 and T4). They are widely distributed in various tissues and organs of the human body and play important roles in processes such as cell growth, differentiation, and metabolic regulation. TRs can inhibit ABCA1 transcription, and TR/RXR heterodimers can bind to the DR-4 element of the ABCA1 promoter. This binding was also confirmed in vivo by Huuskonen et al. through chromatin immunoprecipitation studies [78]. IGF-1 is a polypeptide hormone with high evolutionary conservation. It shares certain homology with insulin in structure and function and plays a core role in processes such as human growth and development, metabolic regulation, and cell survival [79]. IGF-1 can interfere with the PI3-K cascade; the specific mechanism involves activating the PI3-K/Akt/FoxO1 pathway to upregulate ABCA1 transcriptional expression. It is thus speculated that inhibiting PI3-K can indirectly regulate ABCA1 expression, such as through LY294002 (a specific inhibitor of PI3-K) [80].

**Figure 2:**
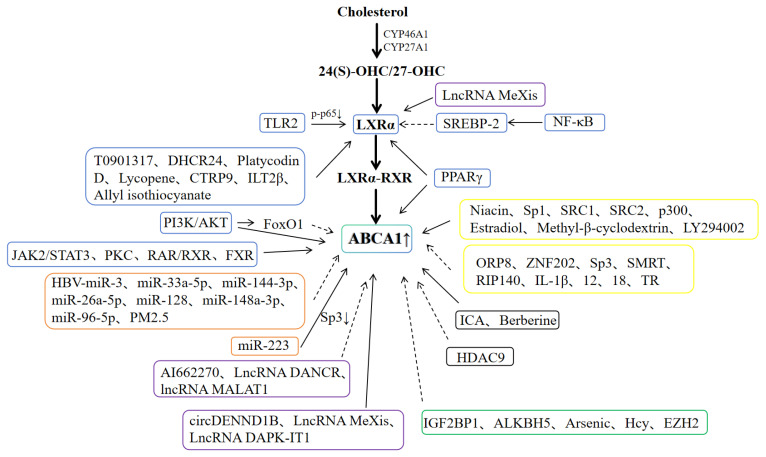
Factors Affecting ABCA1 Transcriptional Expression. Solid lines indicate the promotion of transcriptional expression, and dashed lines indicate the inhibition of transcriptional expression. The blue box represents factors related to signaling pathways, the orange box represents factors related to MicroRNAs, the purple box represents factors related to Non-Coding RNAs, the green box represents factors related to methylation, the black box represents factors related to acetylation, and the yellow box represents other related factors.

In summary, ABCA1 is a key molecule for maintaining lipid homeostasis and regulating reverse cholesterol transport (RCT), and its transcriptional expression is coordinately regulated by multi-dimensional and multi-mechanism pathways, as shown in Figure 2 and Table 1. (1) Signaling pathways constitute the core regulatory network. Dominated by the LXRα/ABCA1 pathway: when intracellular free cholesterol levels increase (e.g., foam cell formation in macrophages), cholesterol is catalyzed by enzymes such as CYP46A1 and CYP27A1 to generate oxysterols, which activate LXRα. Activated LXRα forms a heterodimer with RXR, and this complex binds to the LXRE sequence in the ABCA1 promoter to initiate transcription. PPARγ can enhance the activity of this pathway either by directly binding to the PPRE of ABCA1 or by upregulating LXRα, while TLR2 and SREBP-2 (under certain conditions) can interfere with the pathway. In addition, the JAK2/STAT3 pathway promotes ABCA1 expression through the binding of STAT3 to the ABCA1 promoter; the NF-κB pathway inhibits ABCA1/G1 by mediating SREBP-2 and miR-33a; the PI3K/AKT pathway exerts bidirectional regulation through the mediating role of FoxO1; and pathways such as PKC and RAR-RXR can also upregulate ABCA1. (2) Non-coding RNAs are involved in fine regulation. miRNAs such as HBV-miR-3 and miR-33a-5p directly target ABCA1 mRNA to inhibit its expression, while miR-223 indirectly promotes ABCA1 expression by inhibiting its target gene Sp3. (3) circRNAs (e.g., circDENND1B, circ8411) bind to miRNAs in a “miRNA sponge” mode, relieving the inhibitory effect of miRNAs on ABCA1. lncRNAs (e.g., MeXis, DAPK-IT1) achieve bidirectional regulation of ABCA1 through interaction with proteins or targeting miRNAs. (4) In terms of methylation: m6A modification mediated by IGF2BP1 and ALKBH5, as well as DNA methylation induced by Hcy and arsenic, all inhibit ABCA1 expression. (5) In terms of acetylation: deficiency of HDAC9 and the effect of indole-3-carboxaldehyde (ICA) can promote histone acetylation, thereby enhancing ABCA1 transcription. (6) Other factors participate in coordinated regulation. Promoter-binding proteins such as ZNF202 and Sp3 inhibit ABCA1, while those like Sp1 and SRC1 promote it. Cytokines (e.g., IL-1β) and hormones (e.g., estradiol and thyroid hormones) also affect ABCA1 transcription through binding to specific elements or mediating molecules.

## 4. Factors Affecting Post-Translational Modification of ABCA1

### 4.1. Ubiquitin-Proteasome/Lysosome System

In addition to the transcriptional level regulation mentioned earlier, post-translational modification, as a key link in protein function regulation, also exerts a significant impact on the stability and localization of ABCA1. Ubiquitin-mediated degradation of ABCA1 is mainly divided into the lysosomal pathway and the proteasomal pathway. Among them, the ubiquitin-proteasome system (UPS) is the core pathway for selective protein degradation in eukaryotic cells, and these two pathways jointly regulate the protein level and function of ABCA1. UBE3A is a functionally active protein that engages in interactions with the E6 oncoproteins encoded by human papillomavirus (HPV) types 16 and 18. Through this binding, it facilitates the breakdown of the p53 protein by leveraging the ubiquitin-proteasome system. Research findings have further demonstrated that UBE3A retains its ability to exert ubiquitination activity even when the viral E6 protein is not present in the cellular environment. In a separate study, Melanie and her colleagues conducted experiments using an *Escherichia coli*-based experimental setup. Their results revealed that ABCA1 undergoes monoubiquitination primarily under the mediation of UBE3A. While monoubiquitination is typically linked to the processes of protein sorting and intracellular transport, the question of whether UBE3A exerts an impact on the subcellular localization of ABCA1 remains unresolved and requires further investigation. Beyond its role in regulating protein transport, monoubiquitination can also act as a molecular signal that enables the conjugation of polyubiquitin chains. This conjugation process is carried out by other E3 ligases, which are commonly referred to as E4 ubiquitin ligases in academic literature. Given this mechanism, it is reasonable to hypothesize that besides UBE3A, a second E3 ligase may be involved in the polyubiquitination of ABCA1 and the subsequent degradation process that follows. [110].

Research investigations have indicated that under normal physiological circumstances, COP9 signalosome subunit 3 (abbreviated as CSN3) forms a stable complex with the ABCA1 protein. When external pro-atherosclerotic stimulants—such as thrombin—are present in the cellular environment, ABCA1 undergoes a phosphorylation-dependent dissociation from CSN3. This dissociation event subsequently triggers the degradation of ABCA1. Notably, the forced overexpression of CSN3 exerts multiple regulatory effects: it can inhibit the thrombin-induced ubiquitination process of ABCA1, prevent the subsequent degradation of ABCA1, restore the efficiency of cholesterol efflux, and ultimately suppress the formation of foam cells [111].

Yin and his research team employed techniques including short hairpin RNA (shRNA)-mediated transfection and co-immunoprecipitation to conduct their experiments. Their findings revealed that targeted inhibition of HUWE1 not only promotes the process of cholesterol efflux but also exerts no impact on cholesterol synthesis. Additionally, this inhibition reduces the ubiquitination level of ABCA1, which in turn enhances the stability of the ABCA1 protein [112]. To explore the potential functional relationship between ABCA1 and TANK-binding kinase 1 (TBK1), Lu carried out relevant investigations. These studies demonstrated that the expression level of ABCA1 exhibits a negative correlation with that of TBK1. Furthermore, overexpression of TBK1 was found to increase the direct binding affinity between ABCA1 and components of the ubiquitin-proteasome system. Notably, treatment with MG132—a well-known proteasome inhibitor—was able to prevent the degradation of ABCA1 that is induced by TBK1 overexpression. Collectively, these experimental results suggest that the TBK1-mediated degradation of ABCA1 may occur through a mechanism that promotes the activation of the ubiquitin-proteasome pathway [113]. Wang et al. conducted studies on HepG2 cells and found that human exposure to cadmium (Cd) can upregulate ABCA1 expression and increase its stability by inhibiting the lysosomal pathway, while downregulating OSBP expression by increasing its ubiquitination [114].

Listerin E3 ubiquitin protein ligase 1, commonly referred to as Listerin, is a functionally crucial member of the E3 ubiquitin ligase family and is characterized by the presence of a typical RING domain [115]. In a relevant study, Cao and his research team demonstrated through their findings that Listerin is capable of binding to ABCA1. Following this binding interaction, Listerin catalyzes the K63-linked polyubiquitination of ABCA1 in a site-specific manner—specifically targeting the lysine residues at positions Lys1884 and Lys1957. This site-specific polyubiquitination event exerts two key effects: first, it inhibits the translocation of ABCA1 away from the cell membrane; second, it promotes the degradation of ABCA1 through the ESCRT/lysosomal pathway. Ultimately, these combined effects contribute to the suppression of atherosclerosis development [116]. It has been reported that advanced glycation end products (AGEs) reduce ABCA1 levels (by 20–30%) in J774 and THP-1 macrophages, and induce higher ABCA1 ubiquitination and a faster protein decay rate. The use of proteasomal and lysosomal inhibitors was found to restore ABCA1 in cells treated with AGE-albumin; however, calpain inhibition failed to upregulate ABCA1. In addition, RAGE knockdown also prevented the AGE-induced reduction of ABCA1. Ultimately, it is speculated that AGE-albumin reduces ABCA1 by accelerating its degradation through the proteasomal and lysosomal systems [117]. The apolipoprotein E4 allele (APOE4) is the strongest genetic risk factor for late-onset AD. APOE4 impairs the recycling of ABCA1 and promotes its transport to lysosomes in astrocytes. The accumulation of oxysterols in APOE4 and AD promotes increased expression of ABCA1 and caveolin-1, leading to the endocytosis and sequestration of ABCA1 in lysosomes and the induction of a dysfunctional lysosomal state, in which ABCA1 cannot be recycled back to the plasma membrane [118].

In addition to the several E3 ligases mentioned above, numerous other ubiquitin ligases have been reported to be involved in the ubiquitination and degradation of ABCA1. For example, cullin 3 has been identified as a cullin-RING ubiquitin E3 ligase that mediates the ubiquitination and degradation of ABCA1, thereby inhibiting cholesterol efflux [119]. The ubiquitin ligase HECTD1 regulates the stability of ABCA1 to affect cholesterol export [120]. The E3 ubiquitin ligases HUWE1 and NEDD4-1 have been reported as essential enzymes that post-translationally regulate ABCA1 protein levels and cellular cholesterol export activity [121].

### 4.2. Calpain-Mediated Degradation Pathway

The degradation of ABCA1 by calpains relies on the recognition of specific sequences: these enzymes can target and bind to the PEST sequence (composed of proline, glutamic acid, serine, and threonine) in ABCA1, and mediate the degradation of ABCA1 by promoting the phosphorylation of this sequence. Among them, Thr-1286 and Thr-1305 are two key phosphorylation sites that mediate this process [122,123]. This degradation pathway exerts distinct physiological effects: calpain-mediated ABCA1 degradation directly reduces the expression level of ABCA1 on the cell surface, thereby inhibiting the downstream biogenesis of HDL. The decreased synthesis of HDL may exacerbate the pathological progression of atherosclerosis. Notably, apolipoprotein A-I (ApoA I) can antagonize the aforementioned degradation process through a specific mechanism: when ApoA I binds to ABCA1, it promotes the dephosphorylation of the PEST sequence in ABCA1, thereby blocking the degradation of ABCA1 by calpains and ultimately enhancing the protein stability and overall expression level of ABCA1 [124].

In addition, the N-methyl-D-aspartate receptor (NMDAR) is also involved in the calpain-mediated regulation of ABCA1, among which NMDAR1 is highly expressed in mouse macrophages. When NMDAR is activated, it leads to an increase in intracellular calcium ion (Ca^2+^) concentration, which in turn accelerates the degradation of ABCA1 by activating calpains. This process not only causes intracellular lipid accumulation but also promotes the secretion of a large number of inflammatory mediators [125]. Currently, strategies to interfere with ABCA1 degradation by regulating calpain activity have become a research focus in the field of atherosclerosis treatment, and multiple studies have confirmed the potential roles of certain substances: for example, piperine [126], zinc ions (Zn^2+^), and the soluble epoxide hydrolase inhibitor TPPU can all effectively inhibit calpain-mediated ABCA1 degradation [127,128].

### 4.3. Phosphorylation

ABCA1 is a constitutively phosphorylated protein, which means that under physiological conditions, this protein undergoes continuous phosphorylation modification. Its phosphorylation process is regulated by a variety of signaling molecules and kinases, specifically manifested as the differential effects of different regulatory pathways on protein stability or degradation processes. Unsaturated fatty acids can indirectly promote the degradation of ABCA1 protein in macrophages by activating phospholipase D (PLD); in this process, protein kinase Cδ (PKCδ) further phosphorylates the serine residues of ABCA1, thereby accelerating the degradation process of this protein [129]. On the other hand, other studies have confirmed that apolipoprotein A-I (apoA-I) can phosphorylate the serine residues of ABCA1 by activating the protein kinase Cα (PKCα) signaling pathway; unlike the effect mediated by PKCδ, this phosphorylation process can significantly enhance the stability of ABCA1 protein, thereby maintaining its biological function [130].

**Figure 3:**
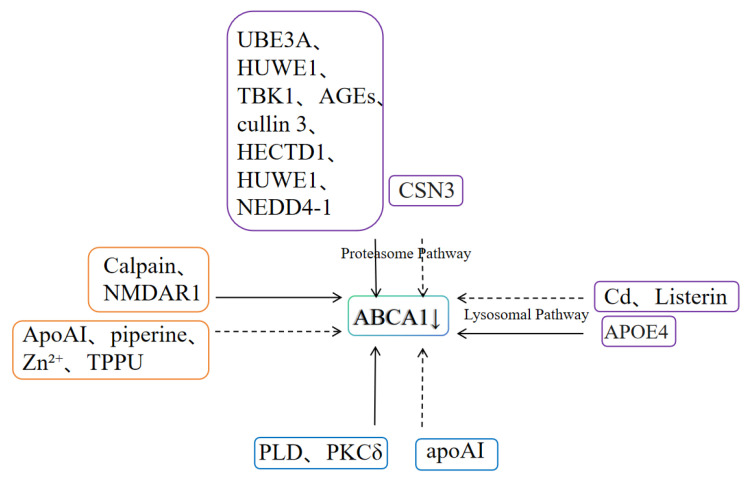
Factors Affecting Post-Translational Modification of ABCA1. Solid lines indicate the promotion of ABCA1 degradation, and dashed lines indicate the inhibition of ABCA1 degradation. The purple box represents factors related to the ubiquitin-proteasome/lysosome system, the orange box represents factors related to the calpain-mediated degradation pathway, and the blue box represents factors related to phosphorylation.

In summary, the post-translational modification of ABCA1 is regulated by three core mechanisms: the ubiquitin-proteasome system, the calpain-mediated degradation pathway, and phosphorylation modification. These mechanisms collectively maintain the homeostasis of ABCA1 protein levels and cholesterol transport function, as shown in Figure 3 and Table 2. In the ubiquitin-proteasome system, multiple E3 ubiquitin ligases such as UBE3A, HUWE1, TBK1, and Listerin mediate monoubiquitination or polyubiquitination modifications, and together with the lysosomal pathway, they regulate ABCA1 degradation. CSN3 can inhibit ABCA1 degradation by binding to it, while substances such as cadmium, AGEs, and APOE4 differentially regulate ABCA1 stability by affecting ubiquitination or lysosomal function, thereby being associated with pathological processes such as atherosclerosis and Alzheimer’s disease (AD). The calpain-mediated degradation pathway relies on the recognition of the PEST sequence in ABCA1. It accelerates ABCA1 degradation by promoting the phosphorylation of this sequence (with key sites at Thr-1286 and Thr-1305), thereby inhibiting HDL biogenesis. ApoA I can antagonize this process by promoting the dephosphorylation of the PEST sequence, while NMDAR accelerates degradation by increasing intracellular Ca^2+^ to activate calpains. Substances such as piperine and Zn^2+^ can inhibit this pathway, making it a research direction for atherosclerosis treatment. Phosphorylation modification exerts “bidirectional regulation” on ABCA1 degradation: unsaturated fatty acids activate PKCδ via PLD, which phosphorylates the serine residues of ABCA1 to accelerate its degradation; in contrast, apoA I activates PKCα to phosphorylate the same residues of ABCA1, but this phosphorylation significantly enhances ABCA1 stability.

## 5. Future Perspectives

Cholesterol is the most abundant sterol in mammalian tissues and performs a variety of important functions in the body. The role of ABCA1 in regulating cholesterol efflux and reducing cholesterol levels is unquestionable, and it is regulated by multiple and highly complex mechanisms. Current research on the regulatory mechanisms of ABCA1 mainly focuses on the modulation of its transcriptional expression. Most studies target its upstream target LXRα to indirectly affect ABCA1 expression—for example, through newly synthesized small-molecule compounds or natural compounds extracted from plants and fruits [81,135,136,137]. Although studies on regulating ABCA1 transcriptional expression through other pathways are also frequently reported, these approaches have not been well applied or promoted. In contrast, far less is known about the post-translational modification mechanisms of ABCA1, and many uncertainties remain regarding the specific processes of its ubiquitination, phosphorylation, and calpain-mediated degradation. Currently, clinically used statins can only moderately increase HDL-C levels (usually by <10%) [138] and fail to improve HDL “function” (such as cholesterol efflux capacity). ABCA1, however, can directly mediate the transport of cholesterol from peripheral cells (e.g., vascular wall macrophages) to HDL precursors (e.g., ApoA1). This process is not only the first step but also the most critical step in HDL-mediated reverse cholesterol transport (RCT). Therefore, investigating the mechanisms underlying ABCA1′ s transcriptional expression and post-translational modification is an indispensable research focus. Studying drugs that target these mechanisms can provide theoretical guidance for drug development and holds significant scientific value and clinical significance for breaking through the bottlenecks of current lipid management, addressing cancer, and preventing cardiovascular diseases (especially atherosclerosis-related diseases). Such research can also help overcome the limitations of existing therapies.

## Figures and Tables

**Table 1:** Factors Related to Affecting ABCA1 Transcriptional Expression.

Types of Regulation	Direct/Indirect	Regulatory Factors	Transactivation/Inhibition	Effects	References
Pathway	Direct	LXRα	Transactivation	Promote Cholesterol Efflux and Treat Hyperlipidemia	[81]
	Direct/Indirect	PPARγ	Transactivation	Promote Cholesterol Metabolism and Treat Alcoholic Fatty Liver	[82]
	Indirect	T0901317	Transactivation	Improve Cholesterol Efflux in Vascular Smooth Muscle Cells	[83]
		TLR2	Transactivation	Promote Airway Smooth Muscle Cell Proliferation and Migration and Inhibit Cell Apoptosis	[84]
		DHCR24	Transactivation	Regulate Lipid Metabolism and Inflammation, and Ameliorate Diseases Such as Atherosclerosis and Cancer	[85]
		SREBP-2	Transactivation	Positively Regulate Cholesterol Efflux	[21]
		Platycodin D	Transactivation	Inhibit the Inflammatory Response of Primary Rat Microglia Stimulated by LPS	[86]
		Lycopene	Transactivation	Regulate Lipid Metabolism to Antagonize DEHP-Induced Hepatotoxicity	[87]
		Allyl isothiocyanate	Transactivation	Activate the LXR Pathway to Reduce Inflammatory Responses and Improve COPD	[24]
		CTRP9	Transactivation	Regulate Macrophage Apoptosis and Cholesterol Reverse Transport	[88]
		LILRB2	Inhibition	Reprogram Cholesterol Metabolism to Drive Gastric Tumorigenesis and Metastasis	[25]
		JAK2/STAT3	Transactivation	Promote ABCA1 Expression in Macrophages to Inhibit Foam Cell Formation	[89]
		NF-κB	Transactivation	Inhibit Lipid Accumulation in Macrophages	[90]
		PI3K/AKT	Inhibition/Transactivation	Regulate Lipid Metabolism	[30,31]
		FXR	Transactivation	Increase Ileal Cholesterol Transport	[17]
MicroRNA	Direct	HBV-miR-3	Inhibition	Promote Cholesterol Accumulation and Facilitate Hepatocellular Carcinoma Progression	[36]
		miR-33a	Inhibition	Promote the Progression of Atherosclerosis	[91]
		miR-144-3p, miR-26a-5p, miR-128, miR-148a-3p, miR-96-5p	Inhibition	Affect Cholesterol Metabolism, Promote the Progression of Atherosclerosis, and Aggravate Bone Loss in Collagen-Induced Arthritis	[40,92,93,94,95]
	Direct/Indirect	miR-223	Inhibition/Transactivation	Regulate Lipid Droplet Clearance in Microglia After Spinal Cord Injury	[41]
	Indirect	miR-200b-3p, miR-424-5p	Inhibition	Promote the Progression of Atherosclerosis	[96,97]
Non-Coding RNA	Direct	circ8411	Transactivation	Alleviate Pyroptosis of Glomerular Endothelial Cells and Improve Diabetic Renal Injury	[47]
		AI662270	Inhibition	Reduce Cholesterol Efflux and Promote Atherosclerosis	[53]
	Indirect	circDENND1B	Transactivation	Promote Cholesterol Efflux and Treat Atherosclerosis	[48]
		LncRNA MeXis	Transactivation	Promote Cholesterol Efflux and Treat Atherosclerosis	[98]
		LncRNA DAPK-IT1	Transactivation	Reduce the Formation of Foam Cells Derived from THP-1 Macrophages	[50]
		LncRNA DANCR	Inhibition	Regulate Lipid Accumulation in Macrophages	[99]
		lncRNA MALAT1	Inhibition	Inhibit Cholesterol Efflux	[100]
Methylation	Direct	IGF2BP	Inhibition	Inhibit Cholesterol Efflux and Promote Lung Adenocarcinoma	[59]
		ALKBH5	Inhibition	Promote Cutaneous Melanoma	[60]
	Indirect	MUC2	Transactivation	Reduce Intracellular Cholesterol Accumulation and Exert Anti-Atherosclerotic Effects	[61]
		Arsenic	Inhibition	Inhibit the Expression of ABCA1 Gene and Protein in Macrophages	[62]
		Hcy	Inhibition	Reduce Cholesterol Efflux and Promote Atherosclerosis	[63]
		EZH2	Inhibition	Reduce Intracellular Cholesterol Efflux, Promote Intracellular Lipid Accumulation and the Progression of Atherosclerosis	[64]
Acetylation	Direct	HDAC9	Inhibition	Inhibit Cholesterol Efflux	[101]
	Indirect	ICA	Transactivation	Promote Cholesterol Efflux	[65]
		Berberine	Transactivation	Promote Cholesterol Efflux	[66]
Other Factors	Direct	Niacin	Transactivation	Promote Cholesterol Efflux	[102]
	Direct	ORP8	Inhibition	Inhibit Cholesterol Efflux	[68]
	Direct	ZNF202	Inhibition	Inhibit Cholesterol Efflux and Promote Atherosclerosis	[103]
	Direct	Sp1/Sp3	Transactivation/Inhibition	Regulate Cholesterol Efflux	[104,105]
	Direct	SRC1, SRC2, p300	Transactivation	Promote Cholesterol Efflux	[71]
	Direct	SMRT, RIP140	Inhibition	Inhibit Cholesterol Efflux	[106,107]
	Indirect	IL-1β, 12, 18	Inhibition	Inhibit Cholesterol Efflux	[73]
	Indirect	Estradiol	Transactivation	Reduce Lipid Content in Hepatocytes	[108]
	Indirect	Methyl-β-cyclodextrin	Transactivation	Reduce Cholesterol Accumulation in Podocytes	[77]
	Indirect	TR	Inhibition	Inhibit Cholesterol Efflux	[109]
	Indirect	LY294002	Transactivation	Promote Cholesterol Efflux	[80]

**Table 2:** Factors Related to Affecting Post-Translational Modification of ABCA1.

Types of Regulation	Direct/Indirect	Regulatory Factors	Transactivation/Inhibition	Effects	References
Ubiquitin-Proteasome/Lysosome System	Direct	UBE3A	Inhibition	Promote Foam Cell Formation and Oppose Myelin Regeneration	[110]
		HUWE1	Inhibition	Regulate Cholesterol Efflux and the Development of Sjögren’s Syndrome	[112]
		TBK1	Inhibition	Retinal Inflammation and Retinal Ganglion Cell Apoptosis	[113]
		cullin 3	Inhibition	Inhibit Cholesterol Efflux and Promote Atherosclerosis	[119]
		HECTD1	Inhibition	Inhibit Cholesterol Efflux	[120]
		NEDD4-1	Inhibition	Inhibit Cholesterol Efflux	[121]
		Listerin	Transactivation	Inhibit Atherosclerosis	[116]
	Indirect	CSN3	Transactivation	Inhibit Foam Cell Formation	[111]
		Cd	Transactivation	Promote Cholesterol Efflux	[114]
		Advanced glycation end products	Inhibition	Inhibit Cholesterol Efflux and Promote Atherosclerosis	[131]
		APOE4	Inhibition	Lipid Metabolism Dysregulation in Alzheimer’s Disease	[132]
Calpain-Mediated Degradation Pathway	Direct	Calpain	Inhibition	Inhibit Cholesterol Efflux and Promote Atherosclerosis	[133]
		ApoA I	Transactivation	Promote Cholesterol Efflux	[124]
	Indirect	NMDAR	Inhibition	Promote Foam Cell Formation	[125]
		Piperine	Inhibition	Promote Foam Cell Formation	[126]
		Zn^2+^	Inhibition	Promote Foam Cell Formation	[127]
		TPPU	Inhibition	Promote Foam Cell Formation	[128]
Phosphorylation	Direct	PKCδ	Inhibition	Inhibit Cholesterol Efflux	[134]
	Indirect	PLD	Inhibition	Inhibit Cholesterol Efflux	[129]
		apoA-I	Transactivation	Promote Cholesterol Efflux	[130]

## Data Availability

No new data were created or analyzed in this study. Data sharing is not applicable to this article.

## References

[1] Zink E., Steinhäuser J., Blickle P.-G., von Meißner W.C.G., Strumann C. (2025). Shift in therapeutic approaches in patients with hypercholesterolemia—A secondary data analysis. BMC Prim. Care.

[2] Aktar A., Vrieze A.M., Telesnicki K., Cox-Duvall P., Arbolino M., DeKoter R.P., Nagpal A.D., Heit B. (2025). GATA2 induces a stem cell-like transcriptional program in macrophages that promotes an atherogenic phenotype. J. Leukocyte Biol..

[3] Feringa F.M., Koppes-den Hertog S.J., Wang L.Y., Derks R.J., Kruijff I., Erlebach L., Heijneman J., Miramontes R., Pömpner N., Blomberg N. (2025). The neurolipid atlas: A lipidomics resource for neurodegenerative diseases. Nat. Metab..

[4] Borràs C., Canyelles M., Santos D., Rotllan N., Núñez E., Vázquez J., Maspoch D., Cano-Sarabia M., Zhao Q., Carmona-Iragui M. (2025). Cerebrospinal fluid lipoprotein-mediated cholesterol delivery to neurons is impaired in Alzheimer’s disease and involves APOE4. J. Lipid Res..

[5] Qiao S., Zou H., Weng Y., Liu Y.F., Li W., Yu X.J., Li L., Zheng L., Xu J. (2025). Targeting SQLE-mediated cholesterol metabolism to enhance CD8+ T cell activation and immunotherapy efficacy in hepatocellular carcinoma. J. Immunother. Cancer.

[6] Jia H.F., Ye X., Zhao Y., Dong L.S. (2023). Mechanisms of Action, Medication Guidance and Adverse Reactions of Statins in the Prevention and Treatment of Strok. Chin. J. Drug Abus. Prev. Treat..

[7] Tang C.K. (2011). Targeting ABCA1 for the Prevention and Treatment of Atherosclerosis. Chin. J. Atheroscler..

[8] An F. (2024). ABCA1Study on the Correlation Between Gene Polymorphism, Methylation Modification and Early-Onset Coronary Heart Disease. Ph.D. Thesis.

[9] Wang S., Smith J.D. (2014). ABCA1 and nascent HDL biogenesis. Biofactors.

[10] Tanaka A.R., Kano F., Ueda K., Murata M. (2008). The ABCA1 Q597R mutant undergoes trafficking from the ER upon ER stress. Biochem. Biophys. Res. Commun..

[11] Neufeld E.B., Remaley A.T., Demosky S.J., Stonik J.A., Cooney A.M., Comly M., Dwyer N.K., Zhang M., Blanchette-Mackie J., Santamarina-Fojo S. (2001). Cellular localization and trafficking of the human ABCA1 transporter*210. J. Biol. Chem..

[12] Linder M.D., Mäyränpää M.I., Peränen J., Pietilä T.E., Pietiäinen V.M., Uronen R.-L., Olkkonen V.M., Kovanen P.T., Ikonen E. (2009). Rab8 regulates ABCA1 cell surface expression and facilitates cholesterol efflux in primary human macrophages. Arter. Thromb. Vasc. Biol..

[13] Lin S., Zhou C., Neufeld E., Wang Y.-H., Xu S.-W., Lu L., Wang Y., Liu Z.P., Li D., Li C. (2013). BIG1, a brefeldin a-inhibited guanine nucleotide-exchange protein modulates ATP-binding cassette transporter a-1 trafficking and function. Arterioscler. Thromb. Vasc. Biol..

[14] Zhao Y.T., Hong L., Jiang H.H., Liu M.M., Zhang H.Z., He L., Chen W.D. (2025). Neuroprotective Effect of Naoluoxintong on Rats After Ischemic Stroke and Its Correlation with LXRα Activation. China Med. Her..

[15] Li N., Wang X., Zhang J., Liu C., Li Y., Feng T., Xu Y., Si S. (2014). Identification of a novel partial agonist of liver X receptor α (LXRα) via screening. Biochem. Pharmacol..

[16] Zhao F., Zhang H., Yu S.H., Liu J.L., Lu M.Y., Zhang J.J. (2025). Effect of Jianpi Qushi Decoction on the PPARγ/LXRα/ABCA1 Pathway in Rats with Dyslipidemia of Spleen Deficiency and Dampness Excess Type. China J. Tradit. Chin. Med. Pharm..

[17] Singh A.B., Dong B., Kraemer F.B., Liu J. (2020). FXR activation promotes intestinal cholesterol excretion and attenuates hyperlipidemia in SR-B1-deficient mice fed a high-fat and high-cholesterol diet. Physiol. Rep..

[18] Gao T., Qian T., Wang T., Su Y., Qiu H., Tang W., Xing Q., Wang L. (2023). T0901317, a liver X receptor agonist, ameliorates perinatal white matter injury induced by ischemia and hypoxia in neonatal rats. Neurosci. Lett..

[19] Zhou E., Ge X., Nakashima H., Li R., van der Zande H.J.P., Liu C., Li Z., Müller C., Bracher F., Mohammed Y. (2023). Inhibition of DHCR24 activates LXRα to ameliorate hepatic steatosis and inflammation. EMBO Mol. Med..

[20] Kim J., Kim J.-Y., Byeon H.-E., Kim J.-W., Kim H.-A., Suh C.-H., Choi S., Linton M.F., Jung J.Y. (2024). Inhibition of toll-like receptors alters macrophage cholesterol efflux and foam cell formation. Int. J. Mol. Sci..

[21] Wong J., Quinn C.M., Brown A.J. (2006). SREBP-2 positively regulates transcription of the cholesterol efflux gene, ABCA1, by generating oxysterol ligands for LXR. Biochem. J..

[22] Hu X., Fu Y., Lu X., Zhang Z., Zhang W., Cao Y., Zhang N. (2016). Protective effects of platycodin D on lipopolysaccharide-induced acute lung injury by activating LXRα-ABCA1 signaling pathway. Front. Immunol..

[23] Mustra Rakic J., Liu C., Veeramachaneni S., Wu D., Paul L., Chen C.-Y.O., Ausman L.M., Wang X.D. (2019). Lycopene inhibits smoke-induced chronic obstructive pulmonary disease and lung carcinogenesis by modulating reverse cholesterol transport in ferrets. Cancer Prev. Res..

[24] Zhu W.-T., Li C.-H., Dai T.-T., Tao F.-L., Wang M.-W., Wang C.-Y., Han Z.L., Sun N.X., Zhao Y.N., Wang D.L. (2021). Effects of allyl isothiocyanate on the expression, function, and its mechanism of ABCA1 and ABCG1 in pulmonary of COPD rats. Int. Immunopharmacol..

[25] Wang X., Liu Y., Zhao Q., Wang X., Chen X., Hou L., Tian S., Peng Z.M., Han X.J., Wang T. (2024). PILRB potentiates the PI3K/AKT signaling pathway and reprograms cholesterol metabolism to drive gastric tumorigenesis and metastasis. Cell Death Dis..

[26] Song X., Liu G., Bin Y., Bai R., Liang B., Yang H. (2025). C1q/tumor necrosis factor-related protein-9 enhances macrophage cholesterol efflux and improves reverse cholesterol transport via AMPK activation. Biochem. Genet..

[27] Mangum L.C., Hou X., Borazjani A., Lee J.H., Ross M.K., Crow J.A. (2018). Silencing carboxylesterase 1 in human THP-1 macrophages perturbs genes regulated by PPARγ/RXR and RAR/RXR: Downregulation of CYP27A1-LXRα signaling. Biochem. J..

[28] Cai Y., Wang Z., Li L., He L., Wu X., Zhang M., Zhu P. (2022). Neuropeptide Y regulates cholesterol uptake and efflux in macrophages and promotes foam cell formation. J. Cell Mol. Med..

[29] Zhao G.-J., Tang S.-L., Lv Y.-C., Ouyang X.-P., He P.-P., Yao F., Tang Y.Y., Zhang M., Tang Y.L., Tang D.P. (2014). NF-κB suppresses the expression of ATP-binding cassette transporter A1/G1 by regulating SREBP-2 and miR-33a in mice. Int. J. Cardiol..

[30] Fukunaga K., Imachi H., Lyu J., Dong T., Sato S., Ibata T., Kobayashi T., Yoshimoto T., Yonezaki K., Matsunaga T. (2018). IGF1 suppresses cholesterol accumulation in the liver of growth hormone-deficient mice via the activation of ABCA1. Am. J. Physiol. Endocrinol. Metab..

[31] Tang Y., Cai Y., Peng F., Li M., Mo Z. (2025). Midkine promote atherosclerosis by regulating the expression of ATP-binding cassette transporter A1 via activator protein-1. Cardiovasc. Drugs Ther..

[32] Nyandwi J.-B., Ko Y.S., Jin H., Yun S.P., Park S.W., Kim H.J. (2021). Rosmarinic acid increases macrophage cholesterol efflux through regulation of ABCA1 and ABCG1 in different mechanisms. Int. J. Mol. Sci..

[33] Bartel D.P. (2004). MicroRNAs: Genomics, biogenesis, mechanism, and function. Cell.

[34] Cifuentes D., Xue H., Taylor D.W., Patnode H., Mishima Y., Cheloufi S., Ma E., Mane S., Hannon G.J., Lawson N.D. (2010). A novel miRNA processing pathway independent of dicer requires Argonaute2 catalytic activity. Science.

[35] Chen X.T., Yang J.B., Zhou Y.Y., Yang S.H., Yang R.H., Fang S.J., Ma Y.X., Niu W.Y. (2025). Research Progress on the Regulatory Mechanisms of 3-Hydroxy-3-Methylglutaryl-Coenzyme A Reductase. Chin. J. Pathophysiol..

[36] Chowdhari S., Deep A., Ahmad B., Samal J., Gupta E., Vivekanandan P. (2025). HBV-miR-3 induces hepatic cholesterol accumulation by targeting ABCA1: Evidence for potential benefits of statin usage. J. Lipid Res..

[37] Zhao T., Li X., Ge Z., Shi J., Wang T., Zhang J., Zhang X., Jiang H., Zhou L., Ye L. (2025). Effects of miR-200b-3p and miR-424–5p regulating notch signaling pathway on atherosclerosis induced by PM2.5 in vitro. Toxicology.

[38] Horie T., Ono K., Horiguchi M., Nishi H., Nakamura T., Nagao K., Kinoshita M., Kuwabara Y., Marusawa H., Iwanaga Y. (2010). MicroRNA-33 encoded by an intron of sterol regulatory element-binding protein 2 (Srebp2) regulates HDL in vivo. Proc. Natl. Acad. Sci. USA.

[39] Goedeke L., Vales-Lara F.M., Fenstermaker M., Cirera-Salinas D., Chamorro-Jorganes A., Ramírez C.M., Mattison J.A., de Cabo R., Suárez Y., Fernández-Hernando C. (2013). A regulatory role for microRNA 33* in controlling lipid metabolism gene expression. Mol. Cell Biol..

[40] Huang K., Pokhrel A., Echesabal-Chen J., Scott J., Bruce T., Jo H., Stamatikos A. (2025). Inhibiting MiR-33a-3p expression fails to enhance ApoAI-mediated cholesterol efflux in pro-inflammatory endothelial cells. Medicina.

[41] Ou Z., Cheng Y., Ma H., Chen K., Lin Q., Chen J., Guo R., Huang Z., Cheng Q., Alaeiilkhchi N. (2024). miR-223 accelerates lipid droplets clearance in microglia following spinal cord injury by upregulating ABCA1. J. Transl. Med..

[42] Jeong S., Jun J.H., Kim J.Y., Park H.J., Cho Y.P., Kim G.J. (2021). Expression of miRNAs targeting ATP binding cassette transporter 1 (ABCA1) among patients with significant carotid artery stenosis. Biomedicines.

[43] Zhu M., Jia L., Jia J. (2021). Inhibition of miR-96-5p may reduce Aβ42/Aβ40 ratio via regulating ATP-binding cassette transporter A1. J. Alzheimer’s Dis. JAD.

[44] Ma L., He S., Li H., Zhang S., Yin Y. (2022). HAND2-AS1 targeting miR-1208/SIRT1 axis alleviates foam cell formation in atherosclerosis. Int. J. Cardiol..

[45] Torres-Paz Y.E., Gamboa R., Fuentevilla-Álvarez G., Cardoso-Saldaña G., Martínez-Alvarado R., Soto M.E., Huesca-Gómez C. (2024). Involvement of expression of miR33-5p and ABCA1 in human peripheral blood mononuclear cells in coronary artery disease. Int. J. Mol. Sci..

[46] Chen W., Wu X., Hu J., Liu X., Guo Z., Wu J., Shao Y., Hao M., Zhang S., Hu W. (2024). The translational potential of miR-26 in atherosclerosis and development of agents for its target genes ACC1/2, COL1A1, CPT1A, FBP1, DGAT2, and SMAD7. Cardiovasc. Diabetol..

[47] Wu W., Wang Y., Shao X., Huang S., Wang J., Zhou S., Liu H., Lin Y., Yu P. (2024). GLP-1RA improves diabetic renal injury by alleviating glomerular endothelial cells pyrotosis via RXRα/circ8411/miR-23a-5p/ABCA1 pathway. PLoS ONE.

[48] Xu F., Shen L., Chen H., Wang R., Zang T., Qian J., Ge J. (2021). circDENND1B participates in the antiatherosclerotic effect of IL-1β monoclonal antibody in mouse by promoting cholesterol efflux via miR-17-5p/Abca1 axis. Front. Cell Dev. Biol..

[49] Sallam T., Jones M., Thomas B.J., Wu X., Gilliland T., Qian K., Eskin A., Casero D., Zhang Z., Sandhu J. (2018). Transcriptional regulation of macrophage cholesterol efflux and atherogenesis by a long noncoding RNA. Nat. Med..

[50] Zhen Z., Ren S., Ji H., Ding X., Zou P., Lu J. (2019). The lncRNA DAPK-IT1 regulates cholesterol metabolism and inflammatory response in macrophages and promotes atherogenesis. Biochem. Biophys. Res. Commun..

[51] Zhao G.-J., Wang Y., An J.-H., Tang W.-Y., Xu X.-D., Ren K. (2024). LncRNA DANCR promotes macrophage lipid accumulation through modulation of membrane cholesterol transporters. Aging.

[52] Fu C.L., Lin Y.M., Tu H.S., Ye J.X., Huang Y.F., Ma D.Z., Zheng C.S. (2024). Mechanism of Tougu Xiaotong Capsules in delaying degeneration of osteoarthritis by regulating cholesterol metabolism in chondrocytes through lncRNA MALAT1. Zhongguo Zhong Yao Za Zhi.

[53] Hong Y., Zhang Y., Chen H., Tang X., Zhao H., Meng Z., Jia X., Liu W., Li X., Wang L. (2023). Genetic dissection of the impact of lncRNA AI662270 during the development of atherosclerosis. J. Transl. Med..

[54] Shen S., Zhang R., Jiang Y., Li Y., Lin L., Liu Z., Zhao Y., Shen H., Hu Z., Wei Y. (2021). Comprehensive analyses of m6A regulators and interactive coding and non-coding RNAs across 32 cancer types. Mol. Cancer.

[55] Lin Y., Li J., Liang S., Chen Y., Li Y., Cun Y., Tian L., Zhou Y., Chen Y., Chu J. (2024). Pan-cancer analysis reveals m6A variation and cell-specific regulatory network in different cancer types. Genom. Proteom. Bioinform..

[56] Zhou E.Y., Yang C., Li W.M., Ran D.C., Xu J.M., Wang C.Q. (2025). m6A Methylation and Osteoporosis. Chin. J. Osteoporos..

[57] Zhang W.H., Jia W.Y., Wang C.R., Jiang Y.J., Yi D., Gong Y.B. (2025). Research Progress of N6-Methyladenine Methylation in Type 2 Diabetes Mellitus. Chin. J. Diabetes.

[58] Wang T., Kong S., Tao M., Ju S. (2020). The potential role of RNA N6-methyladenosine in cancer progression. Mol. Cancer.

[59] Xu S., Liu K., Chen Z., Tang W., Chen Z. (2025). IGF2BP1-mediated methylation of ABCA1 facilitates tumor progression by affecting cholesterol metabolism in lung adenocarcinoma. Amino Acids.

[60] Wang H., Zhao S., Liu H., Liu Y., Zhang Z., Zhou Z., Wang P., Qi S., Xie J. (2024). ALKBH5 facilitates the progression of skin cutaneous melanoma via mediating ABCA1 demethylation and modulating autophagy in an m6A-dependent manner. Int. J. Biol. Sci..

[61] Zhang Y.K. (2025). Valeric Acid Regulates MUC2, Downregulates ALKBH5, Promotes m6A Modification of ABCA1 mRNA, and Alleviates Atherosclerosis. Master’s Thesis.

[62] Zhou T. (2019). Study on the Relationship Between Arsenic Exposure and DNA Methylation in the Promoter Region of ABCA1 Gene. Master’s Thesis.

[63] Wang H.Y., Zhang Z.J., Yuan F.Y., Zeng W.Q., Ma X., Zhai X.J., Gao S.L. (2018). Effect of Homocysteine on Promoter Methylation Level and Expression of ATP-Binding Cassette Transporter A1 in Human Aortic Smooth Muscle Cells. China Med. Her..

[64] Tang Y.Y. (2015). EZH2 Mediates DNA and Histone Methylation in the ABCA1 Promoter Region to Promote the Progression of Atherosclerosis. Master’s Thesis.

[65] Luo W., Meng J., Yu X.H., Zhang Z.Z., Wang G., He J. (2024). Indole-3-carboxaldehyde inhibits inflammatory response and lipid accumulation in macrophages through the miR-1271-5p/HDAC9 pathway. J. Cell Mol. Med..

[66] Deng X., Yi K., Tu J., Li F.J., Chen W.J., Xiao X.H., Jiang Z.S., Tang C.K. (2012). Berberine Regulates Deacetylation of Liver X Receptor α to Promote ATP-Binding Cassette Transporter A1 Expression and Cholesterol Efflux in THP-1 Macrophages. Chin. J. Arterioscler..

[67] Zhang L.H., Kamanna V.S., Ganji S.H., Xiong X.M., Kashyap M.L. (2012). Niacin increases HDL biogenesis by enhancing DR4-dependent transcription of ABCA1 and lipidation of apolipoprotein a-I in HepG2 cells. J. Lipid Res..

[68] Yan D., Mäyränpää M.I., Wong J., Perttilä J., Lehto M., Jauhiainen M., Kovanen P.T., Ehnholm C., Brown A.J., Olkkonen V.M. (2008). OSBP-related protein 8 (ORP8) suppresses ABCA1 expression and cholesterol efflux from macrophages. J. Biol. Chem..

[69] Langmann T., Schumacher C., Morham S.G., Honer C., Heimerl S., Moehle C., Schmitz G. (2003). ZNF202 is inversely regulated with its target genes ABCA1 and apoE during macrophage differentiation and foam cell formation. J. Lipid Res..

[70] Langmann T., Porsch-Ozcürümez M., Heimerl S., Probst M., Moehle C., Taher M., Borsukova H., Kielar D., Kaminski W.E., Dittrich-Wengenroth E. (2002). Identification of sterol-independent regulatory elements in the human ATP-binding cassette transporter A1 promoter: Role of Sp1/3, E-box binding factors, and an oncostatin M-responsive element. J. Biol. Chem..

[71] Wang D., Yeung A.W.K., Atanasov A.G. (2022). A review: Molecular mechanism of regulation of ABCA1 expression. Curr. Protein Pept. Sci..

[72] Hu X., Li S., Wu J., Xia C., Lala D.S. (2003). Liver X receptors interact with corepressors to regulate gene expression. Mol. Endocrinol..

[73] Khovidhunkit W., Moser A.H., Shigenaga J.K., Grunfeld C., Feingold K.R. (2003). Endotoxin down-regulates ABCG5 and ABCG8 in mouse liver and ABCA1 and ABCG1 in J774 murine macrophages: Differential role of LXR. J. Lipid Res..

[74] Thottakkattumana Parameswaran V., Hild C., Eichner G., Ishaque B., Rickert M., Steinmeyer J. (2022). Interleukin-1 induces the release of lubricating phospholipids from human osteoarthritic fibroblast-like synoviocytes. Int. J. Mol. Sci..

[75] Schmitz G., Langmann T. (2005). Transcriptional regulatory networks in lipid metabolism control ABCA1 expression. Biochim. Biophys. Acta..

[76] Kato T., Kawahito H., Kishida S., Irie D., Wakana N., Kikai M., Takata H., Ogata T., Ueyama T., Matoba S. (2015). Bone marrow angiotensin AT2 receptor deficiency aggravates atherosclerosis development by eliminating macrophage liver X receptor-mediated anti-atherogenic actions. J. Renin-Angiotensin-Aldosterone Syst..

[77] Yang Y., Yang Q., Yang J., Ma Y., Ding G. (2017). Angiotensin II induces cholesterol accumulation and injury in podocytes. Sci. Rep..

[78] Huuskonen J., Vishnu M., Pullinger C.R., Fielding P.E., Fielding C.J. (2004). Regulation of ATP-binding cassette transporter A1 transcription by thyroid hormone receptor. Biochemistry.

[79] Zhang Z.R., Yang Y. (2025). Roles of Insulin-Like Growth Factor-1 and Inflammatory Factors in Diabetic Peripheral Neuropathy of Type 2 Diabetes Mellitus. Chin. J. Microcirc..

[80] Lyu J., Imachi H., Iwama H., Zhang H., Murao K. (2016). Insulin-like growth factor 1 regulates the expression of ATP-binding cassette transporter A1 in pancreatic beta cells. Horm. Metab. Res..

[81] Chen X., Yang J., Zhou Y., Wang Q., Xue S., Zhang Y., Niu W. (2025). Research progress and prospects of flavonoids in the treatment of hyperlipidemia: A narrative review. Molecules.

[82] Liu R., Liu P., Li Z., Luo C.Y., Li Z.T., Kang Y. (2024). Effect of Zhige Baogan Jiangzhi Formula on Cholesterol Metabolism in Rats with Alcoholic Liver Disease by Regulating the PPARα-LXRα-ABCA1 Signaling Pathway. Chin. Tradit. Pat. Med..

[83] Borràs C., Rotllan N., Griñán R., Santos D., Solé A., Dong C., Zhao Q., Llorente-Cortes V., Mourín M., Soto B. (2025). Restoring cholesterol efflux in vascular smooth muscle cells transitioning into foam cells through liver X receptor activation. Biomed. Pharmacother..

[84] Wang Y., Yang H., Su X., Cao A., Chen F., Chen P., Yan F., Hu H. (2022). SREBP2 promotes the viability, proliferation, and migration and inhibits apoptosis in TGF-β1-induced airway smooth muscle cells by regulating TLR2/NF-κB/NFATc1/ABCA1 regulatory network. Bioengineered.

[85] Müller C., Hank E., Giera M., Bracher F. (2022). Dehydrocholesterol reductase 24 (DHCR24): Medicinal chemistry, pharmacology and novel therapeutic options. Curr. Med. Chem..

[86] Fu Y., Xin Z., Liu B., Wang J., Wang J., Zhang X., Wang Y., Li F. (2017). Platycodin D inhibits inflammatory response in LPS-stimulated primary rat microglia cells through activating LXRα-ABCA1 signaling pathway. Front. Immunol..

[87] Bao R.K. (2022). Study on Lycopene Antagonizing DEHP-Induced Hepatotoxicity by Regulating Lipid Metabolism. Master’s Thesis.

[88] Lei S.Y. (2021). Study on the Role and Mechanism of CTRP9 in Macrophage Apoptosis and Cholesterol Reverse Transport. Ph.D. Thesis.

[89] Fu H., Tang Y.Y., Ouyang X.P., Tang S.L., Su H., Li X., Huang L.P., He M., Lv Y.C., He P.P. (2014). Interleukin-27 inhibits foam cell formation by promoting macrophage ABCA1 expression through JAK2/STAT3 pathway. Biochem. Biophys. Res. Commun..

[90] Huang J.Y., Jiang X., Jiang C.R., Gong H.Q. (2020). Peroxiredoxin 2 Inhibits Macrophage Lipid Accumulation via the ROS-NF-κB-miR-33a-ABCA1 Pathway. Chin. J. Biochem. Mol. Biol..

[91] Oladosu O., Chin E., Barksdale C., Powell R.R., Bruce T., Stamatikos A. (2024). Inhibition of miR-33a-5p in macrophage-like cells In vitro promotes apoAI-mediated cholesterol efflux. Pathophysiology..

[92] Hu Y.W., Hu Y.R., Zhao J.Y., Li S.F., Ma X., Wu S.G., Lu J.B., Qiu Y.R., Sha Y.H., Wang Y.C. (2014). An agomir of miR-144-3p accelerates plaque formation through impairing reverse cholesterol transport and promoting pro-inflammatory cytokine production. PLoS ONE.

[93] Aryal B., Singh A.K., Rotllan N., Price N., Fernández-Hernando C. (2017). MicroRNAs and lipid metabolism. Curr. Opin. Lipidol..

[94] Kinoo S.M., Chuturgoon A.A., Singh B., Nagiah S. (2021). Hepatic expression of cholesterol regulating genes favour increased circulating low-density lipoprotein in HIV infected patients with gallstone disease: A preliminary study. BMC Infect. Dis..

[95] Wang T., Mo L., Ou J., Fang Q., Wu H., Wu Y., Nandakumar K.S. (2022). Proteus mirabilis vesicles induce mitochondrial apoptosis by regulating miR96-5p/Abca1 to inhibit osteoclastogenesis and bone loss. Front. Immunol..

[96] Zhang Z.Z., Chen J.J., Deng W.Y., Yu X.H., Tan W.H. (2021). CTRP1 decreases ABCA1 expression and promotes lipid accumulation through the miR-424-5p/FoxO1 pathway in THP-1 macrophage-derived foam cells. Cell Biol. Int..

[97] Wu Y.T., Guo Z.J., Li J.B., Ye Z.R., Zhang G.X., Hong C.M., Li M., Wang X.W., Xu W.F., Liang G.T. (2023). Role of MEG3 in cellular physiology of atherosclerosis. Ann. Clin. Lab. Sci..

[98] Zhang S., Li L., Wang J., Zhang T., Ye T., Wang S., Xing D., Chen W. (2021). Recent advances in the regulation of ABCA1 and ABCG1 by lncRNAs. Clin. Chim. Acta; Int. J. Clin. Chem..

[99] Li M.Q. (2021). Study on Long Non-Coding RNA DANCR Regulating Macrophage Lipid Accumulation via miR-33a. Master’s Thesis.

[100] Liu L., Tan L., Yao J., Yang L. (2020). Long non-coding RNA MALAT1 regulates cholesterol accumulation in ox-LDL-induced macrophages via the microRNA-17-5p/ABCA1 axis. Mol. Med. Rep..

[101] Wang Y., Song Z.Y., Ai W.M., Cai J.X. (2024). miR-193a-5p Inhibits Lipid Accumulation in Macrophages via the HDAC9-ABCA1/G1 Pathway. Acta Biochim. Et Biophys. Sin..

[102] Gaidarov I., Chen X., Anthony T., Maciejewski-Lenoir D., Liaw C., Unett D.J. (2013). Differential tissue and ligand-dependent signaling of GPR109A receptor: Implications for anti-atherosclerotic therapeutic potential. Cell Signal..

[103] Peng L., Zhang Z., Zhang M., Yu X., Yao F., Tan Y., Liu D., Gong D., Chong H., Liu X. (2016). Macrophage-activating lipopeptide-2 downregulates the expression of ATP-binding cassette transporter A1 by activating the TLR2/NF-κB/ZNF202 pathway in THP-1 macrophages. Acta Biochim. Biophys. Sin..

[104] Kim J.S., Jung Y.H., Lee H.J., Chae C.W., Choi G.E., Lim J.R., Kim S.Y., Lee J.E., Han H.J. (2021). Melatonin activates ABCA1 via the BiP/NRF1 pathway to suppress high-cholesterol-induced apoptosis of mesenchymal stem cells. Stem Cell Res. Ther..

[105] Nguyen M.-A., Hoang H.-D., Rasheed A., Duchez A.-C., Wyatt H., Cottee M.L., Graber T.E., Susser L., Robichaud S., Berber İ. (2022). miR-223 exerts translational control of proatherogenic genes in macrophages. Circ. Res..

[106] Wagner B.L., Valledor A.F., Shao G., Daige C.L., Bischoff E.D., Petrowski M., Jepsen K., Baek S.H., Heyman R.A., Rosenfeld M.G. (2003). Promoter-specific roles for liver X receptor/corepressor complexes in the regulation of ABCA1 and SREBP1 gene expression. Mol. Cell Biol..

[107] He Y., Zhang L., Li Z., Gao H., Yue Z., Liu Z., Liu X., Feng X., Liu P. (2015). RIP140 triggers foam-cell formation by repressing ABCA1/G1 expression and cholesterol efflux via liver X receptor. FEBS Lett..

[108] Ibata T., Lyu J., Imachi H., Fukunaga K., Sato S., Kobayashi T., Saheki T., Yoshimura T., Murao K. (2022). Effects of 2-methoxyestradiol, a main metabolite of estradiol on hepatic ABCA1 expression in HepG2 cells. Nutrients.

[109] Neggazi S., Canaple L., Hamlat N., Gauthier K., Samarut J., Bricca G., Aouichat-Bouguerra S., Beylot M. (2018). Thyroid hormone receptor alpha deletion in ApoE^-/-^ mice alters the arterial renin-angiotensin system and vascular smooth muscular cell cholesterol metabolism. J. Vasc. Res..

[110] Loix M., Vanherle S., Bolkaerts L., Verberk S.G.S., Punt M., Wouters F., Moonen B., Verhagen R., Van Wouw S.A.E., Jongejan A. (2025). UBE3A promotes foam cell formation and counters remyelination by targeting ABCA1 for proteasomal degradation. Nat. Commun..

[111] Boro M., Govatati S., Kumar R., Singh N.K., Pichavaram P., Traylor J.G., Orr A.W., Rao G.N. (2021). Thrombin-Par1 signaling axis disrupts COP9 signalosome subunit 3-mediated ABCA1 stabilization in inducing foam cell formation and atherogenesis. Cell Death Differ..

[112] Yin J., Xu J., Chen C., Ma X., Zhu H., Xie L., Wang B., Shao Y., Zhao Y., Wei Y. (2023). HECT, UBA and WWE domain containing 1 represses cholesterol efflux during CD4+ T cell activation in sjögren’s syndrome. Frontiers.

[113] Li L., Xu L., Chen W., Li X., Xia Q., Zheng L., Duan Q., Zhang H., Zhao Y. (2018). Reduced annexin A1 secretion by ABCA1 causes retinal inflammation and ganglion cell apoptosis in a murine glaucoma model. Front. Cell Neurosci..

[114] Wang Y., Ji X., Dai S., Liu H., Yan D., Zhou Y., Gu J., Shi H. (2018). Cadmium induced redistribution of cholesterol by upregulating ABCA1 and downregulating OSBP. J. Inorg. Biochem..

[115] Filbeck S., Cerullo F., Pfeffer S., Joazeiro C.A.P. (2022). Ribosome-associated quality-control mechanisms from bacteria to humans. Mol. Cell.

[116] Cao L., Zhang J., Yu L., Yang W., Qi W., Ren R., Liu Y., Hou Y., Cao Y., Li Q. (2025). E3 ubiquitin ligase listerin regulates macrophage cholesterol efflux and atherosclerosis by targeting ABCA1. J. Clin. Investig..

[117] Iborra R.T., Machado-Lima A., Okuda L.S., Pinto P.R., Nakandakare E.R., Machado U.F., Correa-Giannella M.L., Pickford R., Woods T., Brimble M.A. (2018). AGE-albumin enhances ABCA1 degradation by ubiquitin-proteasome and lysosomal pathways in macrophages. J. Diabetes Complicat..

[118] Wang S., Li B., Li J., Cai Z., Hugo C., Sun Y., Qian L., Tcw J., Chui H.C., Dikeman D. (2025). Cellular senescence induced by cholesterol accumulation is mediated by lysosomal ABCA1 in APOE4 and AD. Mol. Neurodegener..

[119] Raghavan S., Singh N.K., Mani A.M., Rao G.N. (2018). Protease-activated receptor 1 inhibits cholesterol efflux and promotes atherogenesis via cullin 3-mediated degradation of the ABCA1 transporter. J. Biol. Chem..

[120] Aleidi S.M., Yang A., Sharpe L.J., Rao G., Cochran B.J., Rye K.-A., Kockx M., Brown A.J., Gelissen I.C. (2018). The E3 ubiquitin ligase, HECTD1, is involved in ABCA1-mediated cholesterol export from macrophages. Biochim. Biophys. Acta. Mol. Cell Biol. Lipids.

[121] Aleidi S.M., Howe V., Sharpe L.J., Yang A., Rao G., Brown A.J., Gelissen I.C. (2015). The E3 ubiquitin ligases, HUWE1 and NEDD4-1, are involved in the post-translational regulation of the ABCG1 and ABCG4 lipid transporters. J. Biol. Chem..

[122] Miyazaki T., Koya T., Kigawa Y., Oguchi T., Lei X.-F., Kim-Kaneyama J., Miyazaki A. (2013). Calpain and atherosclerosis. J. Atheroscler. Thromb..

[123] Yokoyama S., Arakawa R., Wu C.-A., Iwamoto N., Lu R., Tsujita M., Abe-Dohmae S. (2012). Calpain-mediated ABCA1 degradation: Post-translational regulation of ABCA1 for HDL biogenesis. Biochim. Biophys. Acta..

[124] Martinez L.O., Agerholm-Larsen B., Wang N., Chen W., Tall A.R. (2003). Phosphorylation of a pest sequence in ABCA1 promotes calpain degradation and is reversed by ApoA-I. J Biol Chem..

[125] Cheng H., Cheng Q., Bao X., Luo Y., Zhou Y., Li Y., Hua Q., Liu W., Tang S., Feng D. (2020). Over-activation of NMDA receptors promotes ABCA1 degradation and foam cell formation. Biochim. Biophys. Acta Mol. Cell Biol. Lipids..

[126] Wang L., Palme V., Rotter S., Schilcher N., Cukaj M., Wang D., Ladurner A., Heiss E.H., Stangl H., Dirsch V.M. (2017). Piperine inhibits ABCA1 degradation and promotes cholesterol efflux from THP-1-derived macrophages. Mol. Nutr. Food Res..

[127] Lu R., Ishikawa T., Tanaka M., Tsuboi T., Yokoyama S. (2021). Zinc increases ABCA1 by attenuating its clearance through the modulation of calmodulin activity. J. Atheroscler. Thromb..

[128] Peng H., Tang J., Zhao S., Shen L., Xu D. (2019). Inhibition of soluble epoxide hydrolase in macrophages ameliorates the formation of foam cells—Role of heme oxygenase-1. Circ. J..

[129] Ku C.S., Rasmussen H.E., Park Y., Jesch E.D., Lee J. (2011). Unsaturated fatty acids repress the expression of ATP-binding cassette transporter A1 in HepG2 and FHs 74 int cells. Nutr. Res..

[130] Yamauchi Y., Hayashi M., Abe-Dohmae S., Yokoyama S. (2003). Apolipoprotein a-I activates protein kinase C alpha signaling to phosphorylate and stabilize ATP binding cassette transporter A1 for the high density lipoprotein assembly. J. Biol. Chem..

[131] Accacha S., Voloshyna I., Kasselman L.J., Mejia-Corletto J., Srivastava A., Renna H.A., De Leon J., Levine R.L., Reiss A.B. (2025). Plasma from type 1 diabetes patients promotes pro-atherogenic cholesterol transport in human macrophages. J. Investig. Med..

[132] Zhang W.X., Yang Y.L., Yu J.X., Jia L.H., Huang H.-C. (2025). APOE4-driven lipid metabolic dysregulation in alzheimer’s disease: Multi-pathway mechanisms and therapeutic perspectives. Biochem. Biophys. Res. Commun..

[133] Li H., Wang M., Qu K., Xu R., Zhu H. (2023). MP allosterically activates AMPK to enhance ABCA1 stability by retarding the calpain-mediated degradation pathway. Int. J. Mol. Sci..

[134] Wang Y., Oram J.F. (2007). Unsaturated fatty acids phosphorylate and destabilize ABCA1 through a protein kinase C delta pathway. J. Lipid Res..

[135] Moon H.R., Yun J.M. (2025). Protective effect of allium hookeri water extract and its main compound, cycloalliin, on foam cell formation in THP-1-derived macrophages. Food Nutr. Res..

[136] Moumou M., Mokhtari I., Harnafi M., Alrugaibah M., Aljutaily T., Alharbi H.F., Alhuwaymil A., Almutairi A.S., Barakat H., Milenkovic D. (2025). Argan fruit polyphenols regulate lipid homeostasis, prevent liver fat accumulation, and improve antioxidant defense in high-calorie diet fed mice: In vivo study and In silico prediction of possible underlying mechanisms. Metabolites.

[137] Zhang Z., Zhou Y., Lv Q., Gao K., Li Z., Miao Q., Shen L. (2024). Gegen qinlian decoction modulates atherosclerosis and lipid metabolism through cellular interplay and signaling pathways. Comb. Chem. High. Throughput Screen..

[138] Roh J.W., Park M.-H., Son J.-W., Bae S. (2025). HM-ROS-OS-2102 Study Investigator Group. Effectiveness and safety of very-low-dose rosuvastatin-ezetimibe therapy in korean patients with dyslipidaemia: A multicentre prospective observational study. Clin. Drug Investig..

